# Preface

**DOI:** 10.1007/s13199-012-0200-4

**Published:** 2012-12-07

**Authors:** Katarzyna Turnau

**Affiliations:** Institute of Environmental Sciences, Jagiellonian University, Gronostajowa 7, Kraków, Poland

This special volume of Symbiosis contains a selection of papers that were presented during the 7th International Symbiosis Society Congress which was entitled “The earth as a vast symbiosphere”, and was held in Kraków, Poland from the 22–28 July, 2012 (http://www.eko.uj.edu.pl/symbiosis/). The Congress was dedicated to three leaders in the symbiosis field who had passed away since the last ISS congress: Lynn Margulis (1938–2011)^1^, Margalith Galun (1927–2012)^2^ and Gopi Krishna Podila (1957–2010)^3^. We have lost three outstanding researchers, educators, organizers and great friends! Douglas Zook, David Richardson, and Francis Martin, respectively, talked about the careers, personalities and achievements of these three remarkable people. All played an important role in the ISS.

Almost 350 scientists from over 30 countries participated in the 7th International Symbiosis Society Congress in Krakow which provided a fruitful venue and atmosphere for stimulating discussions that covered a wide diversity of symbioses and the mechanisms involved in the interplay between the associating organisms. Plenary presentations were given by: Manuela Giovanetti (Italy) who reported on the “Flow of nutrients and information in Mycorrhizal networks”. She talked of the mechanisms of self-recognition between compatible hyphae, the genetic exchange between different individuals and the formation of interconnecting nets between diverse plants; Frank Ryan (UK) focused on symbiotic viruses and the concept of the “holobiontic genom” showing that endogenous viruses can play an important role in evolution and physiology. Franz Oberwinkler (Germany) talked about the Sebacinales, a very unusual group of fungi that mostly grow as endophytes or form mycorrhizae. A special evening lecture was given by Sieglinde Ott (Germany) who spent several summers in Antarctica studying lichens, their ecology and evolution in this ecosystem. This lecture gave the audience a chance to enjoy wonderful pictures of the scenery and lichens and gain a sense of the unique atmosphere of this continent with its severe climate.

Several symposia were included in the meeting. The symposium entitled “Symbiosis as a driving force” was divided into several sessions. The first was chaired by James A. Lake (USA) who talked on “Using genomes to track the evolution of life on earth”. The second session on “Co-evolutionary mechanisms” was led by Monika Bright (Austria), and these were followed by others on “Horizontal gene transfer and the role of viruses” by Takema Fukatsu (Japan) and “genomics in evolution” by Francis Martin (France).

The second symposium on “Cellular interactions” involved two sessions “integrative processes” chaired by Abdelaziz Heddi (France) and “Recognition systems” by Spencer Nyholm (USA). The symposium on Ecological implications of symbiosis was again devided into several sessions: “Stress physiology” chaired by Hakan Wallander (Sweden), “Ecosystem interactions” by Simon R. Dunn (Australia), “Climate change and environmental impacts” by Rusty Rodriguez (USA). Another session on “Interdisciplinary approaches” included a session on “symbiotic networks and their role in resiliency and vulnerability” chaired by Marcel van der Heijden (Switzerland) and another on “Revealing earth biodiversity” by Franois Lutzoni (USA).

The conference also included applied sessions that focused on “Bioremediation and restoration” chaired by, “Agricultural and industrial enhancement” by Silvio Gianinzzi (France), “Symbiont based control of arthropod pests and disease vectors” by Kostas Bourtzis (Greece) and “Medical implications of symbiosis” chaired by Christain U. Riedel (Germany) and Larry Forney (USA). Due to the great interest in the centres of biological diversity in the tropics, separate session was organized on “Tropical symbioses”, chaired by Ingrid Kottke (Germany).

A very important session/workshop, a follow-up to that presented at previous ISS congresses, was the one organized by Marc-Andre Selosse (France). This concerned symbiosis education. The workshop offered hands-on access to a broad range of symbioses presented by specialists (Fig. 1, 2, 3, 4, 5). The workshop was aimed at researchers, graduate students, and advanced undergraduate students. In different parts of the room, those attending had the opportunity to see and learn how to present the different types of symbioses which experience had shown to be valuable in laboratory classes.

**Fig. 1:**
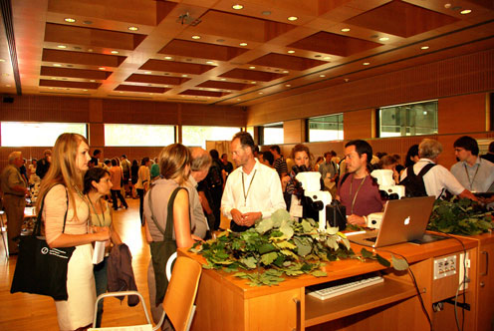
Marc- Andre Selosse, (centre) co-organizer of the workshop on Symbiosis Education with Douglas Zook, talks about and demonstrates domatia to Congress participants (photo by Przemyslaw Ryszka)

**Fig. 2:**
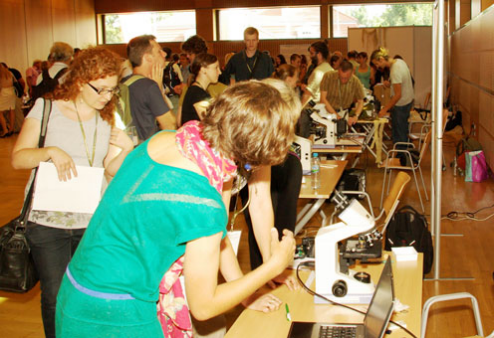
Participants in the workshop on Symbiosis Education examine specimens under the microscope (photo by Przemyslaw Ryszka)

**Fig. 3:**
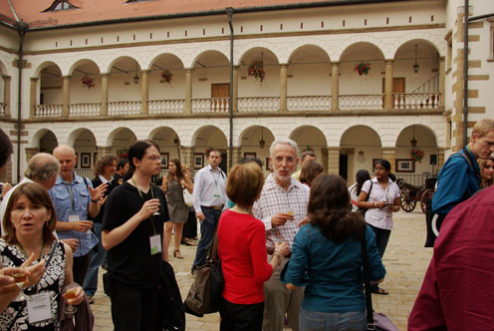
Daniele Armaleo in the checked shirt, a member of the Symbiosis Editorial Board, talks to ISS congress members at the reception prior to the Congress Banquet (photo by Przemyslaw Ryszka)

**Fig. 4:**
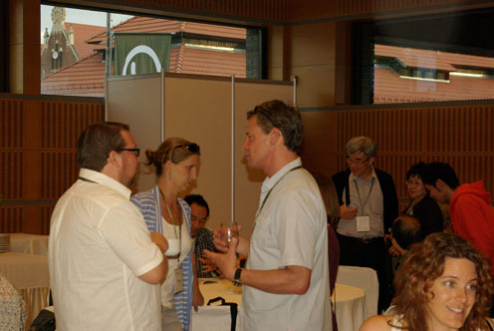
Session leaders, Spencer Nyholm (left) and Simon Dunn (right) discussing at the Congress venue (photo by Przemyslaw Ryszka)

**Fig. 5:**
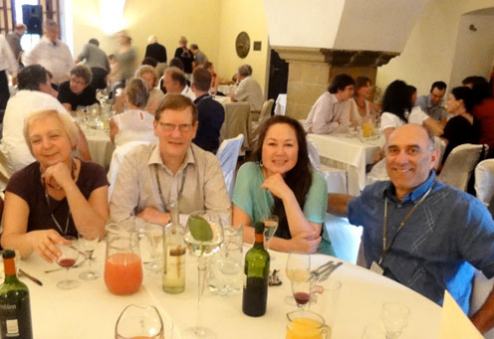
The ISS congress banquet—Left to Right: Katarzyna Turnau, organizer of the 7th international symbiosis congress; David Richardson Editor-in-Chief symbiosis journal; Regina Redman, treasurer of the international symbiosis society, and Rusty Rodriguez President of the international symbiosis society

The stimulating presentations of the Keynote Speakers and the excellent oral contributions of experts from all over the world made this meeting a great event. It was made even more enjoyable by the many contributed posters, most of whose authors were young graduate students filled with knowledge and enthusiasm about their own symbiotic system. The buzz of conversation and discussion was a feature of the poster sessions. The ancient city of Krakow provided a wonderful backdrop to the Congress and field trips were organized to tourist attractions in the surrounding countryside that included a castle and national park which provided participants with information on the local landscape, flora and geology. The Conference Banquet was a highlight being organized in at the Niepolomice Castle, a summer palace a short distance outside Krakow.

The organizers of the conference lead by Katarzyna Turnau (Poland) and Douglas Zook (USA) are grateful to the National Science Foundation (USA), the Polish Ministry for Science and Technology and the US Consulate in Krakow for financial support. The polish team involved many local helping hands and the advice and help of Douglas Zook as a result of his previous experience as a congress organizer was very much appreciated. He spent extended periods in Krakow prior to the Congress and contributed in a very major way to the smooth-running organization and an outstandingly successful congress.

## Footnotes

1 Dolan M, Zook D (2012) Lynn Margulis (1938–2011). Symbiosis 56(1):1–5

2 Balaban M (2012) Margalith Galun (1927–2012). Symbiosis 56(3):149–155

3 Rodriquez R, Zook D (2010) Dr Gopi Krishna Podila (1957–2010). Symbiosis 50(3):117–119

